# The biomass accumulation and nutrient storage of five plant species in an *in*-*situ* phytoremediation experiment in the Ningxia irrigation area

**DOI:** 10.1038/s41598-019-47860-8

**Published:** 2019-08-06

**Authors:** Chongjuan Chen, Fang Wang, Yu Hong, Ruliang Liu, Liangguo Luo

**Affiliations:** 1 Agricultural Clean Watershed Group, Institute of Environment and Sustainable Development in Agriculture, CAAS, Key Laboratory for Agricultural Environment MOA, Engineering & Technology Research Center for Agricultural Non-point Source Pollution Control, Beijing, 100081 China; 2 Institute of Surface-Earth System Science, Tianjin University, Tianjin, 300072 China; 3 Institute of Agricultural Resources and Environment, Ningxia Academy of Agriculture and Forestry Sciences, Yinchuan, 750002 China

**Keywords:** Environmental impact, Plant biotechnology

## Abstract

Phytoremediation has been widely used and is considered an environmentally friendly and efficient method for mitigating nitrogen (N) and phosphorus (P) loads. However, the technique is rarely employed in the Ningxia irrigation area, which suffers from serious N and P pollution. To investigate ways of protecting the aquatic environment in this region, we conducted *in*-*situ* experiments along an agricultural ditch in 2014 and 2015. During the pre-experiment in 2014, five single species floating-bed systems (*Zizania latifolia*, *Oryza sativa*, *Ipomoea aquatica*, *Lactuca sativa* and *Typha latifolia*) and one multi-species floating-bed system with three replicates were evaluated over about two months. *I*. *aquatica* performed best with respect to biomass accumulation and nutrient storage among all plant systems. Multi-species system was not superior to single species systems: 42% and 37% of the N and P storage in the multi-species system were achieved by *I*. *aquatica*. In the formal experiment during 2015, *I*. *aquatica* was tested again and performed excellently with respect to biomass production (1.06 kg/m^2^), N (27.58 g/m^2^) and P (2.34 g/m^2^) uptake. Thus, this study demonstrated that *I*. *aquatica* could be used to reduce N and P loads under saline and alkaline conditions in the Ningxia irrigation area.

## Introduction

Agricultural non-point sources are regarded as important contributors to elevated nitrogen (N) and phosphorus (P) pollution in surface waters; which mainly caused by excessive application and the low use efficiency of fertilizers and pesticides in agricultural ecosystems^1–5^. Agricultural non-point sources (ANPS) contributed 60–87% and 70% of the watershed pollutants in North America^6^ and Sweden^7^, respectively. In China, which is characterized by its agriculture and high fertilizer use, 53% of N loads and 85% of P loads in rivers were derived from ANPS^8^. Furthermore, the losses of N and P to surface water bodies are mainly from rice cultivation, in which both fertilizer and water require huge investment. Thus, excessive N and P pollution in China, where rice is a primary and common food crop, has attracted much attention^9–11^. Many researchers have tried to find some techniques to control and mitigate N and P pollutions^12,13^.

Among these techniques, phytoremediation had been regarded as an effective and environmentally friendly biological method^14^. In phytoremediation, plants not only directly absorb pollutants (such as NO_3_^−^, NH_4_^+^ and PO_4_^3−^) themselves, but may also enhance the removal of pollutants by supporting microbial growth and control algal growth by competing for light and nutrients^15–19^. Phytoremediation may also bring economic benefits by generating by-products^20–24^. So far, many plant species have been used for N and P removal, including *Acorus calamus*, *Canna indica*, *Eichhornia crassipes*, *Ipomoea aquatica*, *Lactuca sativa*, *Lythrum salicaria*, *Oenanthe javanica*, *Oryza sativa*, *Phragmites australis*, *Sagittaria sagittifolia*, *Thalia dealbata*, *Typha latifolia* and *Zizania latifolia*^25–27^.

In addition to Japan^1^, the United States^13,25^ and Australia^28^, phytoremediation has also been reported to be widely applied in China^29,30^, and primarily in southern China because that the rice cultivation is mainly located in southern areas. For example, Kumwimba *et al*.^22^ found significant differences between the vegetated and unvegetated ditches and discussed the importance of plants in nutrient removal in Sichuan Basin. In addition, the study reported that the removal rates of total nitrogen (TN) and total phosphorus (TP) could reach 64% and 58% in a vegetated constructed ditch, and that *C*. *indica* and *O*. *javanica* exhibited higher nutrient storage than other plant species. Wang *et al*.^23^ showed that uptake by *E*. *crassipes* was the primary route for reductions in TN and NH_4_^+^ concentrations, and that this process could remove 113 t N/km^2^ in Lake Caohai. Bu and Xu^18^ suggested that *C*. *indica* was better than other three species for governing eutrophic water om Chongqing. Wang *et al*.^31^ reported the potential of *E*. *crassipes* and *Lolium perenne* in cleaning N and P from Guxin River in Hangzhou. Duan *et al*.^29^ observed that the removal efficiency of TP reached 78.9% and the uptake by *O*. *javanica* accounted for 68.2% of the total removal when the TP concentration was 0.9 mg/L in the Tai Lake region. However, phytoremediation has rarely been applied in northern China, including the Ningxia irrigation area except the researches of Chen *et al*.^20^ and Liu *et al*.^32^.

The Ningxia irrigation area is located in the upper and middle reaches of the Yellow River and is an important area for rice production. According to the Ningxia statistical yearbook^33^, in the recent ten years (from 2008 to 2017), the sown area of rice (8 × 10^4^ ha) averagely accounts for 6.5% of the total sown areas of farm crops (124 × 10^4^ ha) at the autonomous region. The worry is that both fertilizers and pesticides are heavily used in this region and the application rates are still increasing. The consumption of fertilizer increased from 0.23 million tonnes in 1978 to 1.07 million tonnes in 2012 and pesticides from 1.6 kilotonnes to 8.1 kilotonnes, respectively^34^. In addition, this region is characterized by serious soil salinization and alkalization due to low precipitation (180–220 mm) and high evaporation (1000–1550 mm), and agricultural irrigation engineering almost relies on the Yellow River. The farmland drainage water including N, P and many salts was discharged directly into the Yellow River without any pre-treatment, posing a great threat to the water quality of the Yellow River^35^. According to the environment bulletin in recent years, the water quality of agricultural ditches is identified as being at “dangerously high levels” generally, which is mainly due to the high permanganate index, and high concentrations of ammonia nitrogen and TP^36^. Hence, these problems need to be addressed with proper measures.

**Figure 1:**
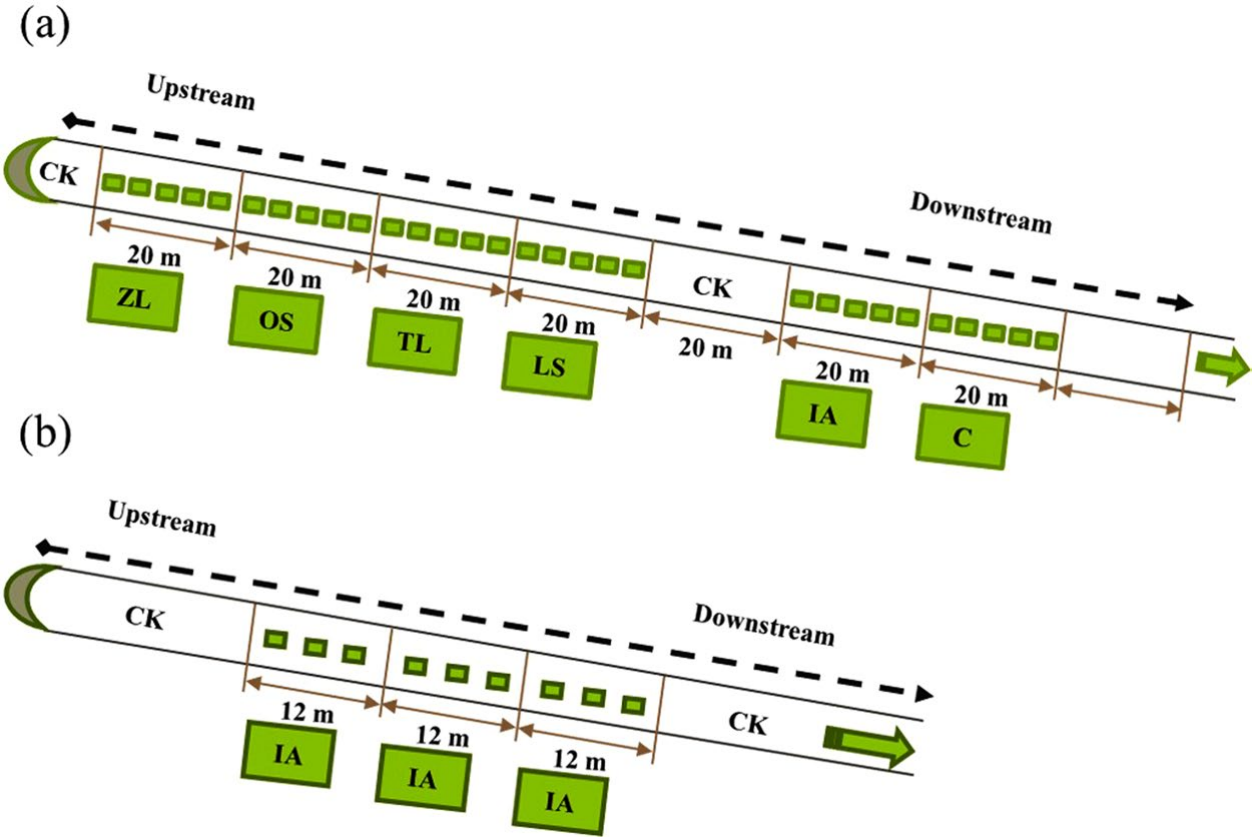
The layout of the vegetated floating-bed systems along the selected representative drainage ditch in the pre-experiment in 2014 (**a**) and the formal experiment in 2015 (**b**). CK represents the control treatment (no plants); ZL, OS, TL, LS, IA and C represent floating-bed systems planted with *Z*. *latifolia*, *O*. *sativa*, *T*. *latifolia*, *L*. *sativa*, *I*. *aquatica* and a mixture of the five plant species, respectively.

Together with the simulation experiment, which we carried out in 2014^20^, a parallel *in*-*situ* experiment was conducted in an agricultural ditch (Fig. 1a). In 2015, we also conducted an *in*-*situ* experiment to confirm the results from 2014 (Fig. 1b). The objective of this study was to identify which plant species are most suitable for controlling N and P pollution under saline and alkaline conditions in the Ningxia irrigation area. Target plant species were evaluated based on their potential for biomass accumulation and nutrient storage.

## Results

### Plant biomass accumulation in the pre-experiment

In the pre-experiment in 2014, the plants grew well and gained high biomass at the end of the experiment, except for OS, which exhibited poor growth. The fresh biomass accumulation of each plant floating-bed treatment is shown in Table 1. Among the five systems, IA had the highest fresh biomass accumulation (844.98 ± 29.41 g/m^2^), followed by the ZL system (649.72 ± 91.25 g/m^2^), while TL had the lowest fresh biomass accumulation (129.33 ± 33.07 g/m^2^). The fresh weight of the multi-species system (C) was moderate (471.82 ± 90.53 g/m^2^). In addition, there was greater biomass accumulation in below-ground parts than in above-ground parts in all floating-bed systems except for LS (Table 1).

**Table 1:** Fresh biomass accumulation in whole plants, above-ground and below-ground parts, and the percent above-ground accumulation in the five types of plant floating-bed systems at the end of the *in*-*situ* experiment in 2014.

Species	Fresh biomass accumulation g/m^2^	Above-ground g/m^2^	Below-ground g/m^2^	Percent above-ground %
ZL	649.72 ± 91.25 bc	185.30 ± 26.02 bc	464.42 ± 65.22 c	28.52 ± 1.98 a
TL	129.33 ± 33.07 a	47.66 ± 12.19 a	81.67 ± 20.88 a	36.85 ± 1.80 bc
LS	199.66 ± 25.85 a	128.68 ± 16.66 ab	70.98 ± 9.19 a	64.45 ± 0.64 d
IA	844.98 ± 29.41 c	298.07 ± 10.38 c	546.91 ± 19.04 c	35.28 ± 1.80 ab
C	471.82 ± 90.53 b	204.05 ± 39.15 bc	267.77 ± 51.38 b	43.25 ± 4.64 c

Note: Values are means ± SE, the same letters (a–d) indicate subsets that are not significantly different within each column (*P* > 0.05). ZL, TL, LS, IA and C represent floating-bed systems planted with *Z*. *latifolia*, *T*. *latifolia*, *L*. *sativa*, *I*. *aquatica* and a mixture of species, respectively.

**Figure 2:**
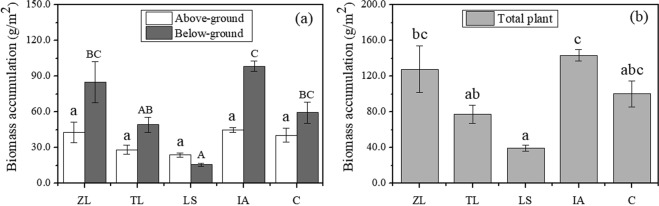
Dry biomass accumulation in above- and below-ground plant parts (**a**) and total plant (**b**) in the five types of floating-bed system at the end of the *in*-*situ* experiment in 2014. Error bars represent standard errors of the biomass accumulation in above-ground (white), below-ground (dark gray) and total plant (light gray). Different letters indicate significant difference at the 0.05 level. ZL, TL, LS, IA and C represent floating-bed systems planted with *Z*. *latifolia*, *T*. *latifolia*, *L*. *sativa*, *I*. *aquatica* and a mixture of species, respectively.

With respect to dry biomass accumulation in the different systems in 2014, the highest weight was recorded for the IA system (143.39 ± 6.62 g/m^2^) and the lowest for the LS system (39.61 ± 3.10 g/m^2^) (Fig. 2b). There were system-specific differences in the location of biomass accumulation, with ZL (85.19 ± 17.29 g/m^2^), TL (49.24 ± 6.33 g/m^2^), IA (98.43 ± 4.55 g/m^2^) and C (59.61 ± 8.85 g/m^2^) mainly accumulating biomass in below-ground parts, while LS (23.86 ± 1.86 g/m^2^) primarily allocated biomass to its above-ground parts (Fig. 2a).

### Plant nutrient storage in the pre-experiment

**Figure 3:**
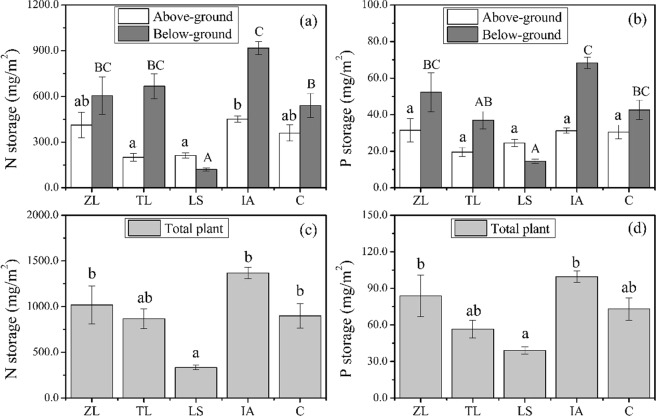
Nitrogen storage in above- and below-ground plant parts (**a**) and total plant (**c**), phosphorus storage in above- and below-ground plant parts (**b**) and total plant (**d**) in the five types of floating-bed systems at the end of the *in*-*situ* experiment in 2014. Error bars represent standard errors for nutrient storage in above-ground (white), below-ground (dark gray) and total plant (light gray). Different letters indicate significant difference at the 0.05 level. ZL, TL, LS, IA and C represent floating-bed systems planted with *Z*. *latifolia*, *T*. *latifolia*, *L*. *sativa*, *I*. *aquatica* and a mixture of species, respectively.

**Figure 4:**
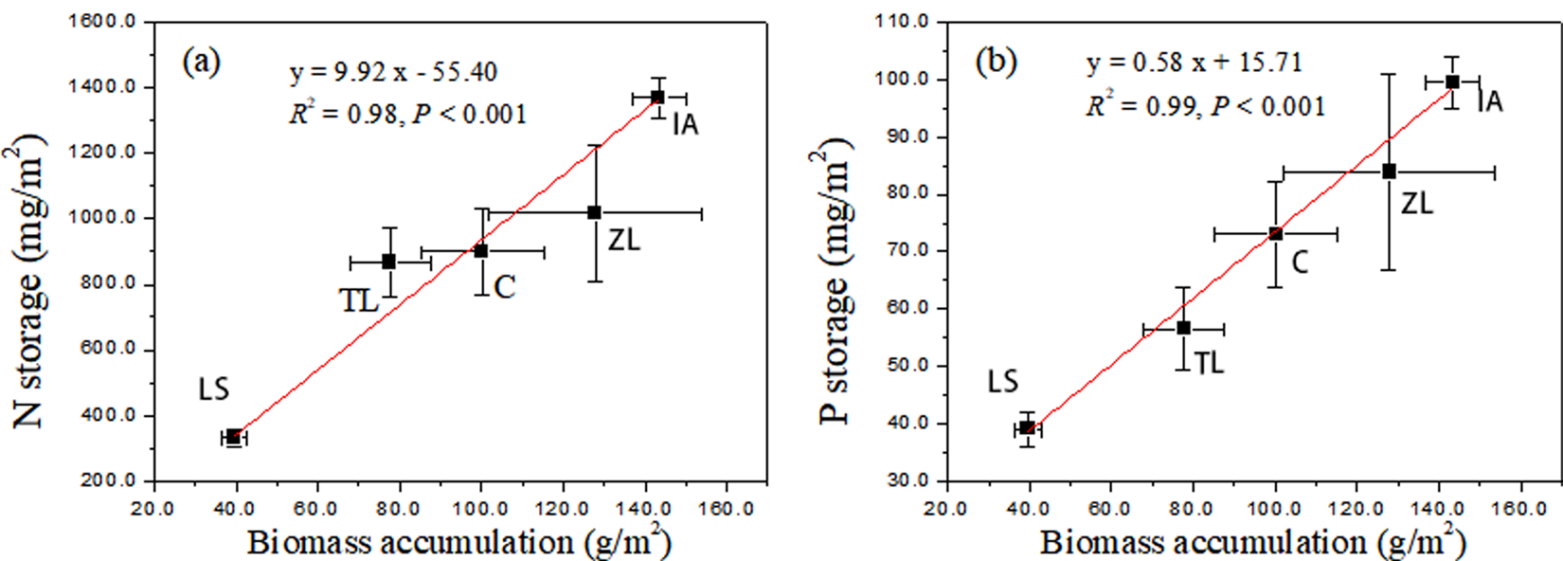
The relationships between nitrogen (**a**) and phosphorus (**b**) storage and dry biomass accumulation at the end of the *in*-*situ* experiment in 2014. ZL, TL, LS, IA and C represent the floating-bed systems planted with *Z*. *latifolia*, *T*. *latifolia*, *L*. *sativa*, *I*. *aquatica* and a mixture of the five plant species, respectively.

In the pre-experiment in 2014, the N content of above-ground was highest in the IA and lowest in the TL system and the N content of below-ground was highest in the TL and lowest in the ZL system (Table 2). For the P content, the highest values both above-ground and below-ground were in the LS system, and the lowest values were recorded in the IA and ZL systems (Table 2). The N and P storage levels per square meter in different floating-bed systems are presented in Fig. 3. The highest N and P total storage values were both in the IA system: 1368.18 ± 63.27 mg/m^2^ and 99.54 ± 4.60 mg/m^2^, respectively (Fig. 3c,d). The lowest N (333.07 ± 25.69 mg/m^2^) and P (39.08 ± 3.03 mg/m^2^) storage values were both recorded in the LS system. Similar to the results for total biomass accumulation, N and P were mostly accumulated below-ground, except in the LS system (Fig. 3a,b). The ranking for N storage was same as that for P storage for all the floating-bed systems, from high to low: IA > ZL > C > TL > LS. In addition, both N and P storage was found to be correlated highly and positively with the biomass accumulation (Fig. 4).

**Table 2:** Nitrogen and phosphorus content in above-ground and below-ground plant tissue at the end of the *in*-*situ* trial in 2014.

Species	TN content (g/kg)		TP content (g/kg)	
	Above-ground	Below-ground	Above-ground	Below-ground
ZL	9.64 ± 0.14 b	7.09 ± 0.17 a	0.74 ± 0.04 a	0.61 ± 0.03 a
TL	6.91 ± 0.27 a	13.28 ± 0.56 c	0.69 ± 0.09 a	0.75 ± 0.07 a,b
IA	10.02 ± 0.51 b	9.32 ± 0.28 b	0.69 ± 0.02 a	0.69 ± 0.03 a
LS	8.89 ± 0.20 b	7.67 ± 0.37 a	1.03 ± 0.06 b	0.93 ± 0.04 b

Note: Values are means ± SE, the same letters (a–d) indicate subsets that are not significantly different within each column (*P* > 0.05). TN and TP represent total nitrogen and total phosphorus, respectively. ZL, TL, IA and LS represent *Z*. *latifolia*, *T*. *latifolia*, *I*. *aquatica* and *L*. *sativa*, respectively.

### Biomass accumulation and nutrient storage in *I*. *aquatica* in the formal experiment

In the formal experiment in 2015, the IA system grew very well and generated more biomass than in 2014. The total fresh and dry biomass accumulation was 17.64 ± 3.62 kg/m^2^ and 1.05 ± 0.21 kg/m^2^, respectively, mainly found in above-ground parts (Table 3). N and P contents in IA were significantly higher than the values in 2014, and higher in the above-ground than the below-ground parts (Table 3). Furthermore, N (27.58 ± 6.97 g/m^2^) and P (2.34 ± 0.57 g/m^2^) storage in IA was obviously greater than in 2014, with nutrient storage above-ground far greater than below-ground (Table 3).

**Table 3:** The fresh and dry biomass accumulation, nitrogen and phosphorus content, nitrogen and phosphorus storage in the *I*. *aquatica* floating-bed treatment in 2015.

	Fresh biomass kg/m^2^	Dry biomass kg/m^2^	TN content g/kg	TP content g/kg	TN storage g/m^2^	TP storage g/m^2^
Above-ground	9.08 ± 1.45	0.69 ± 0.12	28.13 ± 1.89	2.20 ± 0.10	19.91 ± 5.14	1.55 ± 0.37
Below-ground	8.56 ± 1.86	0.36 ± 0.07	21.03 ± 0.97	2.14 ± 0.07	7.67 ± 1.84	0.79 ± 0.20

Note: Values are means ± SE. TN and TP represent total nitrogen and total phosphorus, respectively.

## Discussion

This study was conducted along an agricultural drainage ditch where various environmental factors could affect plant N and P uptake. In the present study, the water temperature and dissolved oxygen (DO) were suitable for plant growth (Table 4). The water conductivity and pH values indicated that soil salinization and alkalization may have resulted in saline and alkaline agricultural drainage water. The water conductivity and pH were lower than the values recorded in the simulation experiment^20^, suggesting that the presence of plants could be used to mitigate alkaline and saline conditions^29^.

**Table 4:** Water quality parameters of the agricultural drainage ditch in 2014.

Items	Values
TN (mg/L)	0.65 ± 0.07
TP (mg/L)	0.06 ± 0.01
Temperature (°C)	21.32 ± 0.18
DO (mg/L)	5.07 ± 0.17
Conductivity (μs/cm)	974.08 ± 3.62
pH	8.10 ± 0.14

Note:Values are means ± SE. TN, TP and DO represent total nitrogen, total phosphorus and dissolved oxygen, respectively.

From upstream to downstream in the experimental drainage ditch, water shields were set to keep the environment between treatment systems relatively independent; there were small outlets on each to facilitate water flow while ensuring enough water retention time to allow the plants to absorb N and P. There were differences in nutrient levels in the ditch between different sampling dates, but in the majority of cases, there was no significant difference in nutrient levels among different plant systems on the same sampling date (Fig. S1). In addition, agricultural drainage ditches have some capacity for purification because of the presence of weeds on the banks and plenty of sediment in the bottom of the ditch. Thus, the differences in plant biomass accumulation and N and P storage among plant floating-bed systems could provide a better demonstration of the differences in nutrient purification capacity among different plant systems than the absolute changes in nutrient concentrations in the ditch. In this case, more attention was focused on biomass accumulation, and N and P storage in plants rather than the change in nutrient concentration in the drainage water.

In phytoremediation systems, the removal of N mainly depends on direct uptake by plants, nitrification-denitrification by microbes, sedimentation and a little ammonia volatilization^1,18,26^. The removal of P is primarily the result of plant uptake, adsorption to the sediment or substrate, physical or chemical precipitation and a little PH_3_ volatilization^15,27,37,38^. For plants that float on the water surface of agricultural drainage ditches, can only absorb nutrients from drainage water to satisfy their growth requirements because there are no other substrates^39,40^. Many previous studies^23,41,42^, as well as our pot experiment^20^, have demonstrated the significant role of plant uptake in phytoremediation. In addition, agricultural drainage ditches possess certain purification capacity because of the presence of weeds on the banks and plenty of sediment in the bottom of the ditch. Thus, in the present study, the differences in plant biomass accumulation and N and P storage among plant floating-bed systems provided a better demonstration of the differences in nutrient purification capacity among different plant systems than absolute decreases in nutrient concentrations in the water.

The plant fresh biomass accumulation in the pre-experiment in 2014 ranged from 129.33 g/m^2^ to 844.98 g/m^2^ (Table 1), these values were lower than results reported by Tanner and Headley (2011) (834~2350 g/m^2^)^43^ and Zhao *et al*. (2012) (255.5~2338.9 g/m^2^)^44^, mainly because of the high plant cover rate and nutrient concentration levels (6.9~8.3 mg/L of TN in Zhao *et al*.^44^) as well as certain substrates supporting good growth^43^. Similarly, the dry biomass accumulation in this study (39.61~143.39 g/m^2^) was a little lower than the data (33.7~307.1 g/m^2^) presented by Zhu *et al*.^45^. This may be because of the higher nutrient concentrations in the water (mean TN and TP: 2.1 mg/L and 0.154 mg/L) and nutrient contents in the plants (15.31~23.15 g/kg in TN and 1.07~1.89 g/kg in TP) reported by Zhu *et al*.^45^. This suggested that plants can grow well and have some biomass accumulation even under low nutrients conditions.

Among the plant species included in our study, the highest dry biomass accumulation was recorded in the IA system; furthermore, the statistical results showed that IA floating-beds accounted for 29% of the biomass accumulation in the multi-species system. All these data suggested that IA has great capacity for nutrient acquisition and biomass accumulation. A number of studies^40,42,46,47^ have reported the value and outstanding characteristics of IA in water purification. In the present study, the planting time of floating-beds was later than the timing of general fertilizer application. Little fertilizer is applied in the later season so there were relatively low N and P concentrations in the drainage ditch (Table 4 and Fig. S1). To better absorb nutrients and support growth, the root system of IA grew larger, and the mean root length reached 47.29 ± 0.78 cm, thus, the below-ground biomass accumulation was higher than that above-ground.

The biomass accumulation of IA in 2015 was higher compared to some previous experiments^43–45^ and the pre-experiment in 2014. Several facts may explain this difference. First, nutrient conditions, hydraulic index and planting density of IA in 2015 were almost the same as in 2014, but there were significantly more weeds growing on the banks of ditch in 2014 than 2015 because we cleared nearly all weeds before the experiment in 2015. Thus, in 2015, under similar nutrients conditions, plants were not forced to compete with weeds for nutrition to maintain growth and could, therefore, accumulate more biomass. Second, the difference in biomass accumulation in IA between 2015 and 2014 might also be explained by inter-annual climate differences. In addition, the IA transplantation time was two weeks earlier in 2014 than 2015, and perhaps the relatively lower temperature was not conducive to IA growth. This highlights the importance of transplantation time.

Most of the N and P absorbed by plants are stored in plant tissues^45,48^. Thus, some studies have reported positive correlations between N and P storage and biomass^45,49^. We also observed that plant N and P storage values were highly correlated with biomass accumulation in 2014. For this reason, N and P storages were ranked the same as biomass accumulation among these plant systems. The N storage ranged from 333.07 mg/m^2^ to 1368.18 mg/m^2^, P storage from 39.08 mg/m^2^ to 99.54 mg/m^2^. IA was the most while LS was the least efficient at nutrient storage (Fig. 3). The accumulated nutrients were lower compared to the results from some previous studies^44,45,50^. For example, Zhu *et al*.^45^ found that N and P contents could reach 15.31~23.15 g/kg and 1.07~1.89 g/kg, respectively. As for specific plants, such as *O*. *javanica*, *Gypsophila* sp. and *Salix babvlonica*, N and P storages of them were 2.17 g/m^2^ and 0.167 g/m^2^, 3.92 g/m^2^ and 0.247 g/m^2^, 4.48 g/m^2^ and 0.331 g/m^2^, respectively; these values were higher than the most efficient plant system in the Ningxia irrigation area. Furthermore, in Zhejiang Province, *T*. *dealbata* also showed excellent nutrient uptake (60.9 g N/m^2^ and 8.2 g P/m^2^) due to high nutrient concentrations in rivers and intensive planting^45^. Thus, low plant N (6.91~13.28 g/kg) and P contents (0.61~1.03 g/kg) were probably attributed to the low nutrient storage values in the Ningxia irrigation area as well as the low nutrient concentrations in ditch water.

In the formal experiment, N and P accumulations in IA reached 27.58 g/m^2^ and 2.34 g/m^2^, respectively (Table 3). The values were higher than the data reported by Zhu *et al*.^45^ and lower than those of Zhao *et al*.^44^. In the formal experiment, N and P were primarily accumulated in the above-ground part of IA. As an edible vegetable, the above-ground part of IA can be harvested about every 15 days. In the Ningxia irrigation area, IA could take up 87.30 g N/m^2^ and 6.99 g P/m^2^ provided that the above-ground material was collected four times during the experiment. This indicated the importance of regular management in the application of phytoremediation. In particular, IA could remove 11.60 kg N/year and 0.98 kg P/year from drainage ditch if IA floating-beds cover the total water surface of this agricultural drainage ditch (850 m in length, 1.5 m in width). The good performance of IA floating-beds in the Ningxia irrigation area illustrated the potential of phytoremediation for the drainage water quality from farmland, and supports the need for large scale tests to evaluate its sustainable treatment performance.

Single plant systems have limitations in terms of nitrogen and phosphorus assimilation, both temporally and spatially, which would have potential effects on N and P removal rates. While multi-species system should deliver good performance by buffering against the variations in weather and nutrient conditions as a result of its diversity and adaptability^51^. Previous studies found that *E*. *crassipes* and *Lemna* spp. in combination with blue-green algae delivered better performance than *E*. *crassipes* alone^24,51^. However, Picard *et al*.^52^ reported that purification efficiency of a combination of four plant species (*Scirpus validus*, *Phalaris arundinacea*, *T*. *latifolia* and *Carex lacustris*) was little different from that of *S*. *validus*, *P*. *arundinacea* and *T*. *latifolia* alone. Lin *et al*.^53^ also found that *Myriophyllum spicatum* and *Hydrocotyle verticillata* outperformed their combination with respect to N and P removal rates in a eutrophic urban river. Like the latter two studies, we observed that combined plant systems did not deliver better performance than single plant systems with respect to biomass accumulation, N and P storage. There are two possible reasons. First, OS did not grow well, this might decrease the overall performance of the multi-species system. Second, all plants used in this experiment were emergent, this kind of combined system could perform less well than the combination of emergent and floating plants since the later could enhance nutrient uptake at a wider spatial scale. In addition, in this study, IA played a significant role in the multi-species system, being responsible for 42% and 37% of the N and P storage.

In conclusion, as suggested by several other studies^46,54,55^, IA not only exhibited strong removal capacity, but also had great advantage in biomass production and nutrient accumulation. In the Ningxia irrigation area, IA outperformed other plant species under saline and alkaline conditions, the harvested plants could be utilized to generate biogas, biomaterial and even food^24^, and it could deliver both economic and ecological benefits. Thus, IA is suitable for reducing nutrient pollution associated with farmland drainage in the Ningxia irrigation area.

## Conclusions

The objective of the present study was to identify the best performing plant species for controlling N and P pollution in the Ningxia irrigation area. *In*-*situ* experiments were conducted along an agricultural ditch in 2014 and 2015. In the pre-experiment in 2014, dry biomass accumulation of different plant floating-bed systems ranged from 39.61 g/m^2^ to 143.39 g/m^2^, nutrient storage increased linearly with biomass accumulation. Plant N and P storage could reach 333.07~1368.18 mg/m^2^ and 39.08~99.54 mg/m^2^, respectively. Among the five kinds of floating-bed systems, IA was the best and LS was the least in terms of biomass production and nutrient assimilation. In addition, multi-species systems had no advantages relative to the single species floating-bed systems with respect to N and P assimilation efficiency; IA was responsible for almost half of the nutrient accumulation in multi-species systems. According to the results of pre-experiment in 2014, IA was tested again in 2015. In the formal experiment in 2015, IA performed excellently in terms of dry biomass production (1.06 kg/m^2^), N (27.58 g/m^2^) and P (2.34 g/m^2^) uptake. Biomass and nutrients were primarily accumulated particularly in above-ground parts. Therefore, among these tested plant species, IA was the most effective and promising species in mitigating N and P pollution in farmland drainage water in the Ningxia irrigation area.

## Materials and Methods

### Experimental set up

The *in*-*situ* phytoremediation experiment examining the removal of N and P was conducted along a 800 m agricultural ditch in Qingtongxia (N 37°36′~38°15′, E 105°21′~106°21′), which is 60 km away from Yinchuan city and is located within the Ningxia irrigation area. We conducted a pre-experiment in 2014 and the formal experiment in 2015. During the rice growing period, the average depth and width of the water in the ditch is 1.0 ± 0.02 m and 1.5 ± 0.04 m, and the average water flow velocity is 0.18 ± 0.05 m/s. The floating-beds used in the experiment were made with PVC tubes and nylon nets. The floating-bed was assembled with two pairs of tubes (length × width: 2 m × 1 m, 10 cm in diameter) and covered with nylon net, the area of each floating-bed was 2 m^2^. The floating-bed was able to support a weight of 100 kg.

In the pre-experiment in 2014, five plant species – *Zizania latifolia* (ZL), *Oryza sativa* (OS), *Ipomoea aquatica* (IA), *Lactuca sativa* (LS) and *Typha latifolia* (TL) – were selected for testing because they could control N and P pollution and the harvested plants could provide edible grains or vegetables^20,25–27^. The seedlings of TL and ZL were collected from local ponds, the seedlings of IA and LS were bought from a local plant nursery. Plants of uniform height (12 cm for IA, 8 cm for LS, and 16 cm for TL and ZL) were selected, one group was carefully washed in tap water and distilled water to remove all the debris and then weighed to determine biomass, the other group was directly transplanted into floating-beds and held in place with a sponge. The planting density of OS was 150 individuals/m^2^, ZL and TL were at a density of 42 individuals/m^2^, LS and IA at 30 individuals/m^2^, respectively.

In the pre-experiment in 2014, there were two methods of floating-bed plant cultivation. One involved single species floating-bed systems (one plant species in each system) with five floating-beds. The other was a multi-species system (five plant species in each system) with three replicates. Each floating-bed plant cultivation system was 20 m long, with 5 floating-beds distributed evenly along the drainage ditch, covering 33.3% of the water surface. A stretch of the ditch with no floating-beds was treated as the control (CK). For the single-species systems, the plants used were ZL, OS, LS, IA and TL. While the multi-species systems (C) involved these five plant species in combination. Thus, the layout of the planting systems from upstream to downstream along the drainage ditch were arranged in the order CK, ZL, OS, TL, LS, CK, IA, C, C, C and CK (Fig. 1a). The pre-experiment was carried out from June 23 to September 1, 2014. All plants species were harvested on September 1, 2014 except for LS which was collected on August 8. However, OS did not seem to be adapted to the nutrient environment in the water body and grew so poorly that it was not harvested.

Based on the results from both pot experiments^20^ and *in*-*situ* pre-experiment, IA was selected for use in the *in*-*situ* drainage ditch experiment in 2015, using seedlings of the same size as those in 2014. The whole experiment involved the CK treatment with two replicates and the IA treatment with three replicates. The length of each plant system was 12 m, with three floating-beds in the IA treatment, the floating-beds covered 33.3% of the water surface. Thus, from upstream to downstream, the treatment arrangement was CK, IA, IA, IA and CK (Fig. 1b). The experimental period ran from July 7 to September 7, 2015.

### Sampling and analyses

In the pre-experiment in 2014, 100 mL water samples were collected from the drainage ditch in the morning every sixth day. In the formal experiment in 2015, samples of the same volume were collected in the morning every tenth day. Basic water quality parameters, including water temperature, pH, water conductivity and dissolved oxygen (DO), were measured *in*-*situ* using a portable meter (YSI Pro Plus, USA). Water samples were then taken to the lab for total nitrogen (TN) and total phosphorus (TP) determination. Total N concentration was determined spectrophotometrically (UV) after alkaline potassium persulfate digestion, and TP concentration was determined using the colorimetric ammonium molybdate method^40^.

At the end of the trial, plant samples were carefully harvested and gently washed with deionized water. Plant samples were divided into the above-ground and below-ground parts, the fresh biomass was measured. Thereafter, the plant samples were dried at 70 °C in an oven to constant dry weight. The Kjeldahl method and the vanadium molybdate yellow colorimetric method were used to determine plant N and P contents, respectively^56^. The plant N and P storage values were calculated by multiplying the N or P content by the dry weight.

### Statistical analyses

Biomass accumulation per square meter was calculated as follows:

1 $${\rm{Biomass}}\,{\rm{accumulation}}({\rm{g}}/{{\rm{m}}}^{{\rm{2}}})=({{\rm{W}}}_{{\rm{t}}}-{{\rm{W}}}_{{\rm{0}}})/{\rm{2A}}$$

where W_0_ is the average dry weight of plants at the beginning of the trial, W_t_ is the dry weight of the harvested plants at the end of the trail, and A is the number of floating-beds in each system.

Nutrient storage per square meter was calculated as follows:

2 $${\rm{Nutrient}}\,{\rm{storage}}({\rm{g}}/{{\rm{m}}}^{{\rm{2}}})={\rm{biomass}}\,{\rm{accumulation}}\times {\rm{nutrient}}\,{\rm{content}}$$

where biomass accumulation is calculated according to Eq. (1) and nutrient content is the TN or TP content of the plant samples.

Data in this study are presented as mean ± standard error. All figures are plotted with origin 8.5. Statistical analyses were conducted using SPSS (SPSS for Windows, Version 20.0, Chicago, IL, USA). Comparisons of biomass accumulation and nutrient storage between different plant systems were conducted using one-way ANOVA, multiple comparisons were performed with post-hoc Tukey tests. Differences between means were deemed significant if *P* < 0.05.

## Supplementary information

supplementary information

## Data Availability

The data will be available online.
