# Supplementary material for: The biomass accumulation and nutrient storage of five plant species in an in-situ phytoremediation experiment in the Ningxia irrigation area
Source: Sci Rep. 2019 Aug 6;9:11365. doi: 10.1038/s41598-019-47860-8 (PMC6684586; doi:10.1038/s41598-019-47860-8)
Supplement: Supplementary file 1 — supplementary information [file 41598_2019_47860_MOESM1_ESM.pdf]

The biomass accumulation and nutrient storage of five plant species in an in-situ phytoremediation experiment in the Ningxia irrigation area

Chongjuan Chen[a,b], Fang Wang[c], Yu Hong [c], Ruliang Liu[c], Liangguo Luo[a*]

[a]Agricultural Clean Watershed Group, Institute of Environment and Sustainable Development in Agriculture, CAAS, Key Laboratory for Agricultural Environment MOA, Engineering & Technology Research Center for Agricultural Non-point Source Pollution Control, Beijing, 100081, China.

[b]Institute of Surface-Earth System Science, Tianjin University, Tianjin, 300072, China.

[c]Institute of Agricultural Resources and Environment, Ningxia Academy of Agriculture and Forestry Sciences, Yinchuan, 750002, China.

*Corresponding author: Liangguo Luo

E-mail address: luoliangguo@caas.cn

Supplementary information

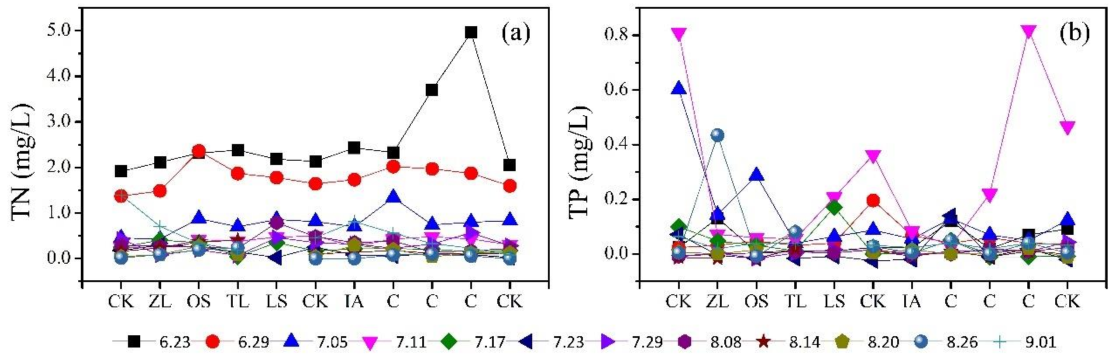

Fig S1. The total nitrogen (a) and total phosphorus (b) concentrations in different plant systems along the ditch on specific sampling dates in the pre-experiment in 2014.
